# Effect of a 12-Week Walking Program Monitored by Global Physical Capacity Score (GPCS) on Circulating Cell-Free mtDNA and DNase Activity in Patients with Irritable Bowel Syndrome

**DOI:** 10.3390/ijms25084293

**Published:** 2024-04-12

**Authors:** Guglielmina Chimienti, Francesco Russo, Antonella Bianco, Fatima Maqoud, Caterina De Virgilio, Grazia Galeano, Antonella Orlando, Giuseppe Riezzo, Benedetta D’Attoma, Antonia Ignazzi, Michele Linsalata, Laura Prospero, Isabella Franco, Claudia Beatrice Bagnato, Ritanna Curci, Sergio Coletta

**Affiliations:** 1 Department of Biosciences, Biotechnologies and Environment, University of Bari Aldo Moro, 70125 Bari, Italy; guglielminaalessandra.chimienti@uniba.it (G.C.); caterina.devirgilio@uniba.it (C.D.V.); 2 Functional Gastrointestinal Disorders Research Group, National Institute of Gastroenterology IRCCS “Saverio de Bellis”, 70013 Castellana Grotte, Italy; fatima.maqoud@irccsdebellis.it (F.M.); grazia.galeano@irccsdebellis.it (G.G.); antonella.orlando@irccsdebellis.it (A.O.); giuseppe.riezzo@irccsdebellis.it (G.R.); benedetta.dattoma@irccsdebellis.it (B.D.); antonia.ignazzi@irccsdebellis.it (A.I.); michele.linsalata@irccsdebellis.it (M.L.); laura.prospero@irccsdebellis.it (L.P.); 3 Laboratory of Movement and Wellness, National Institute of Gastroenterology IRCCS “Saverio de Bellis”, 70013 Castellana Grotte, Italy; antonella.bianco@irccsdebellis.it (A.B.); isabella.franco@irccsdebellis.it (I.F.); claudia.bagnato@irccsdebellis.it (C.B.B.); ritanna.curci@irccsdebellis.it (R.C.); 4 Core Facility Biobank, National Institute of Gastroenterology IRCCS “Saverio de Bellis”, 70013 Castellana Grotte, Italy; sergio.coletta@irccsdebellis.it

**Keywords:** cf-mtDNA, DNase activity, IBS, exercise, inflammation, Global Physical Capacity core

## Abstract

Irritable bowel syndrome (IBS) involves low-grade mucosal inflammation. Among the various approaches capable of managing the symptoms, physical activity is still under investigation. Despite its benefits, it promotes oxidative stress and inflammation. Mitochondria impacts gut disorders by releasing damage-associated molecular patterns, such as cell-free mtDNA (cf-mtDNA), which support inflammation. This study evaluated the effects of a 12-week walking program on the cf-mtDNA and DNase in 26 IBS and 17 non-IBS subjects. Pro- and anti-inflammatory cytokines were evaluated by ELISA. Digital droplet PCR was used to quantify cf-mtDNA; DNase activity was assessed using a single radial enzyme diffusion assay. PCR-RFLP was used to genotype *DNASE1* rs1053874 SNP. Significantly lower IL-10 levels were found in IBS than in non-IBS individuals. Exercise reduced cf-mtDNA in non-IBS subjects but not in IBS patients. DNase activity did not correlate with the cf-mtDNA levels in IBS patients post-exercise, indicating imbalanced cf-mtDNA clearance. Different rs1053874 SNP frequencies were not found between groups. The study confirms the positive effects of regular moderate-intensity physical activity in healthy subjects and its role in cf-mtDNA release and clearance. Walking alone might not sufficiently reduce subclinical inflammation in IBS, based on imbalanced pro- and anti-inflammatory molecules. Prolonged programs are necessary to investigate their effects on inflammatory markers in IBS.

## 1. Introduction

Irritable bowel syndrome (IBS) is a functional disorder characterized by sensory and motor dysfunctions in the gastrointestinal (GI) tract, typically without structural abnormalities. This syndrome can cause recurring symptoms, impacting the patient’s quality of life and healthcare expenses. Depending on the patient’s bowel habits, IBS is divided into four types: IBS-D (diarrhea-predominant), IBS-C (constipation-predominant), IBS-M (mixed type), and IBS-U (unspecified) [1].

The evidence of alterations in the intestinal barrier, especially in patients suffering from IBS-D, with modifications in the small intestinal permeability, has narrowed the definition of IBS as an actual functional disease [2]. A dysfunctional intestinal barrier could be the origin or lead to the persistent, low-grade mucosal inflammation observed in the course of IBS [3,4] as it happens in other inflammatory GI diseases (e.g., inflammatory bowel disease (IBD) [5]) and celiac disease (CD) [6].

Emerging evidence supports the broad role of mitochondria in several inflammatory and malignant GI disorders [7,8,9]. Mitochondria lie at the center of mucosal inflammation, owing to their central roles in energy production, which is fundamental for maintaining intestinal barrier function, metabolism, cell division, maturation, and death [10]. Dysfunctional mitochondria are likely to fuel gut mucosal pathogenesis [11]. Mitochondria play pivotal roles in cellular oxidative stress, being the source and the target of a major amount of free reactive species [12]. Cellular oxidative stress causes mitochondrial injury, triggering the production of mitochondrial damage-associated molecular patterns (mtDAMPS), such as oxidized cardiolipin, N-formyl peptides, extracellular ATP, and mtDNA release into the cytoplasm and extracellular space as cell-free mtDNA (cf-mtDNA), which in turn perpetuate inflammatory tissue damage, acting as “sterile” mediators of inflammation [13]. mtDNA is a central factor in the activation of an immune response involving the intracellular Toll-like receptor 9 (TLR9), expressed in several immunological and non-immunological cells [14], due to its hypomethylated feature, similar to its bacterial ancestor, the NLRP3 inflammasome, and the cGAS pathways [15].

Cell-free nucleic acids (cf-NAs) are any nucleic acids present outside the cell in various biological fluids, such as plasma, urine, saliva, and stool. They are considered valuable biomarkers for several conditions, such as cancer and cardiovascular and autoimmune diseases [16,17]. Cell-free DNA (cf-DNA) of both nuclear (cf-nDNA) and mitochondrial origins is released into circulation after apoptosis, necrosis, and active secretion from cells as part of the physiological cell turnover or pathology [18]. Its composition and quantity dramatically change during the pathological conditions relevant to inflammatory processes. Indeed, cell-free DNA is also released from neutrophils during infection or inflammation as neutrophil extracellular traps (NETs), DNA webs interspersed with granular and histone proteins, acting as immobilizers and killers of pathogens [19]. Circulating cf-DNA and NETs are mainly cleared by Deoxyribonuclease (DNase) I, and a deficiency in this enzyme, which leads to the persistence of circulating DNA/chromatin complexes, has been associated with the development of chronic inflammation and subsequent autoimmune diseases [20].

Pharmacological treatments and psychological interventions address specific symptoms of IBS well, so the research for a combination of standard therapies and complementary treatments is still a growing field. Beneficial alternative treatments include dietary approaches, such as a low FODMAPs (fermentable oligo-, di-, mono-saccharides, and polyols) diet [21,22] or an alternative grain-based diet [23]. According to the multifactorial pathogenesis of the disease, the management of patients can also include encouraging lifestyle modifications and increasing physical activity (PA). Although some previous studies provided low certainty of evidence [24], several reports highlighted the beneficial effects of PA, especially walking and therapeutic yoga, in reducing IBS severity and improving several symptoms [25]. Despite its beneficial effects, depending on its duration and intensity, PA also promotes hemostatic perturbations, such as thermal, metabolic, and mechanic stress, leading to increased oxidative stress and inflammation [26] driven by myokines, such as IL-6, that are secreted into the blood by muscle cells during heavy exercise [27]. Of note, the importance of performing adequate exercise, in terms of type, frequency, and intensity, has been assessed by the WHO, at least in the case of peculiar groups, such as older subjects [28]. Indeed, cf-DNA is quantitatively released into the plasma in the non-pathological setting of acute and intense physical exercise, thus activating the innate immune response [29]. However, several reports have provided evidence that regular PA, in turn, promotes the removal of cf-DNA, thereby lowering pro-inflammatory signaling [30,31].

Our previous papers have demonstrated the positive impact of a twelve-week walking program as a moderate-intensity aerobic exercise on the gastrointestinal and psychological profiles in IBS patients [32,33]. To shed more light on the relationship between the health benefits of exercise and IBS, we evaluated cf-mtDNA levels before and after twelve weeks of moderate exercise and the activity of its endogenous counter-regulator, DNase I. Since the human *DNASE1* gene is polymorphic, we genotyped the non-synonymous rs1053874 SNP, which is involved in the susceptibility to common diseases [34], in subjects under investigation. Therefore, we determined the number of copies/μL of plasma cf-mtDNA by digital droplet PCR (ddPCR), evaluated serum DNase activity by a single radial enzyme diffusion assay, and genotyped the rs1053874 SNP by the PCR-RFLP method in both IBS patients and non-IBS controls that underwent the three-month walking program. The effects of PA on physical capacity, as a predictor of response to the exercise program, were assessed using the Global Physical Capacity Score (GPCS).

## 2. Results

**Figure 1:**
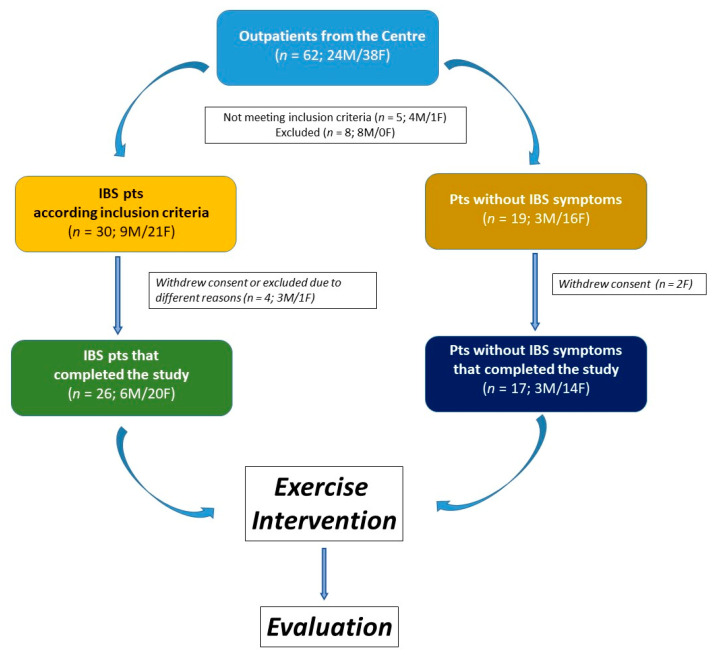
The flowchart of the study.

The study included 43 participants, 26 of whom were classified as IBS patients. The remaining 17 subjects were classified as non-IBS subjects. They did not exhibit lower gut symptoms but reported mild symptoms of upper gut diseases, such as dyspepsia or gastroesophageal reflux. The study flow is illustrated in Figure 1.

### 2.1. Patient Characteristics

The participants’ characteristics are presented in Table 1. Gender composition, age, and BMI were not significantly different between the groups. After twelve weeks of a moderate aerobic exercise program, IBS patients and non-IBS subjects showed a reduced BMI. The decrease was statistically significant in both IBS patients (*p* = 0.0258, paired *t*-test) and non-IBS subjects (*p* = 0.0033, paired *t*-test). No significant differences were found between the groups as concerns the IPAQ.

**Figure 2:**
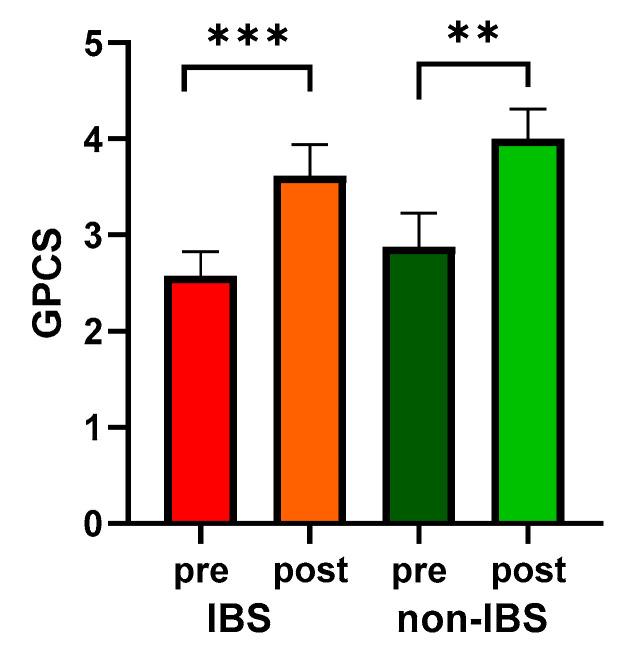
GPCS values in 26 IBS patients and 17 non-IBS subjects. Bars represent mean and SEM. ***: *p* < 0.001; **: *p* < 0.01 (Wilcoxon matched-pairs signed-rank test). GPCS = Global Physical Capacity Score; IBS = irritable bowel syndrome; pre and post = before and after 12 weeks of exercise program.

Moreover, both groups showed significantly enhanced PC, quantified as GPCS, compared to their baseline values (2.58 ± 0.25 vs. 3.62 ± 0.32, *p* < 0.0001, and 2.88 ± 0.35 vs. 4.00 ± 0.31, *p* = 0.0078 in IBS and non-IBS group, respectively; Wilcoxon matched-pairs signed-rank test). On the other hand, no significant differences in GPCS were found between the two groups either before or after physical exercise (Figure 2).

### 2.2. Effects of Walking on the Cytokine Profile

The inflammatory profile in the two study groups was analyzed by assessing the serum concentrations of the pro-inflammatory IL-6, IL-8, and TNF-alpha, and the anti-inflammatory IL-10 cytokines (Table 2). At baseline, no significant difference was observed in the concentration of the pro-inflammatory cytokines between the two groups. Conversely, the anti-inflammatory IL-10 was significantly lower in the IBS group than in the non-IBS one (*p* = 0.04776; Mann–Whitney test). After the exercise program, the two groups did not show significant differences in any of the cytokines under investigation. 

### 2.3. Effects of Walking on Plasma cf-mtDNA Concentration

**Figure 3:**
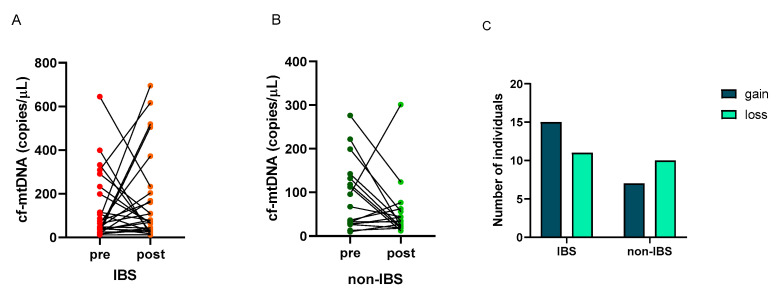
Individual cf-mtDNA response to the twelve-week walking program. cf-mtDNA copies/μL were determined in pre- and post-exercise plasma samples. Lines represent individual values in the 26 IBS patients (Panel (**A**)) and 17 non-IBS subjects (Panel (**B**)). Panel (**C**): distribution of IBS patients and non-IBS subjects according to cf-mtDNA variation after exercise. IBS = irritable bowel syndrome; pre and post = before and after 12 weeks of the exercise program; gain = increased cf-mtDNA copies/μL at the end of walking program; loss = decreased cf-mtDNA copies/μL at the end of the program.

The number of copies/μL of cf-mtDNA was determined in the plasma of the subjects before and after the walking program. A great individual variability appeared in the cf-mtDNA response to exercise as follows: 15 (58%) and 11 (42%) IBS patients showed an increase (gain) or a decrease (loss) in this parameter, respectively. On the contrary, 7 (41%) and 10 (59%) non-IBS subjects showed an increase (gain) or a decrease (loss), respectively (Figure 3A,B). No statistically significant difference was found in the distribution of individuals who experienced gain or loss between IBS patients and non-IBS ones (*p* = 0.3580; Fisher’s exact test) (Figure 3C).

**Figure 4:**
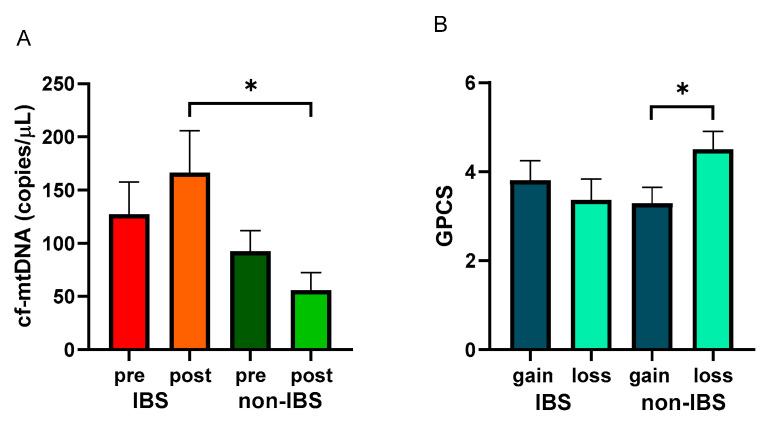
Levels of cf-mtDNA and GPCS values with regard to cf-mtDNA variations. Panel (**A**): cf-mtDNA copies/μL were determined in pre- and post-exercise plasma samples from 26 IBS patients and 17 non-IBS subjects. *: *p* < 0.05, Mann–Whitney test. Panel (**B**): GPCS values in 26 IBS patients and 17 non-IBS subjects dichotomized according to gain or loss of cf-mtDNA after the exercise program. *: *p* < 0.05, unpaired *t*-test. GPCS = Global Physical Capacity Score; IBS = irritable bowel syndrome; pre and post = before and after 12 weeks of exercise program; gain = increased cf-mtDNA copies/μL at the end of the walking program; loss = decreased cf-mtDNA copies/μL at the end of walking program.

After the walking program, the IBS patient group showed a non-significant increase in cf-mtDNA copies/μL (127.30 ± 30.32 vs. 166.40 ± 39.51 copies/μL, as pre- and post-values, respectively; *p* = 0.5651, Wilcoxon matched-pairs signed-rank test), whereas non-IBS subjects group showed a non-significant reduction (92.49 ± 19.54 vs. 55.82 ± 16.75 copies/μL, as pre and post values, respectively; *p* = 0.2247, Wilcoxon matched-pairs signed-rank test). Indeed, a statistically significant difference was found between the post-exercise values of the two groups (*p* = 0.0268; Mann–Whitney test) (Figure 4A).

GPCS values were analyzed in the IBS and non-IBS groups and dichotomized based on cf-mtDNA gain or loss after the training. Non-IBS subjects that showed a loss of cf-mtDNA after exercise reached statistically significantly higher GPCS than their counterpart (4.00 ± 0.31 vs. 2.88 ± 0.35, as loss and gain values, respectively; *p* = 0.0494, unpaired *t*-test). IBS patients showed a non-significant difference between the dichotomized groups (3.62 ± 0.32 vs. 2.58 ± 0.25, as loss and gain values, respectively; *p* = 0.5167; unpaired *t*-test) (Figure 4B).

### 2.4. Effects of Walking on Serum DNase Activity

**Figure 5:**
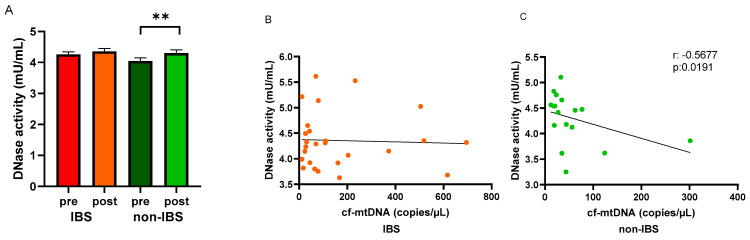
Levels of DNase activity and regression analyses. Panel (**A**): DNase activity (mU/mL) was determined in pre- and post-exercise serum samples from the 26 IBS patients and 17 non-IBS subjects. **: *p* < 0.01, paired *t*-test. Panel (**B**): correlation analysis between cf-mtDNA and DNase activity in the 26 IBS patients after the exercise program. Panel (**C**): correlation analysis between cf-mtDNA and DNase activity in the 17 non-IBS subjects after the exercise program. r, *p*: Spearman’s R correlation test. IBS = irritable bowel syndrome; pre and post = before and after 12 weeks of exercise program.

Circulating DNase activity was evaluated in the study groups before and after the walking program. An increased DNase activity was observed in the serum of both IBS patients and non-IBS subjects after the program compared to baseline levels. The increase was statistically significant in non-IBS subjects (4.05 ± 0.10 vs. 4.30 ± 0.10 mU/mL, as pre- and post-values, respectively; *p* = 0.0137, paired *t*-test) but not in IBS patients (4.26 ± 0.08 vs. 4.36 ± 0.10 mU/mL, as pre and post values, respectively; *p* = 0.7561, paired *t*-test) (Figure 5A). In search of a correlation between cf-mtDNA and the circulating waste-management enzyme after regular PA, Spearman’s R correlation test was applied to the IBS and non-IBS group values. A significant inverse correlation was found in the non-IBS group (r: −0.5677, *p* = 0.0191) (Figure 5C) but not in the IBS group (r: −0.03658, *p* = 0.8592) (Figure 5B).

### 2.5. Frequency Distribution of DNASE1 Gene Polymorphism and Effects of Genetics on DNase1 Activity

The rs1053874 SNP in the *DNASE1* gene was genotyped in the study groups. The distribution of genotypes and allele frequencies is presented in Table 3. The polymorphic distribution was found in Hardy–Weinberg equilibrium (*p* = 0.97581, total sample; *p* = 0.898564, IBS group; *p* = 0.900569, Non-IBS group; χ2 test). The G allele was shown to be predominant. No statistically significant differences were observed in genotype (*p* = 0.9429, χ2 test) or allele (*p* = 0.8223, Fisher’s exact test) distributions between the IBS and non-IBS groups.

**Figure 6:**
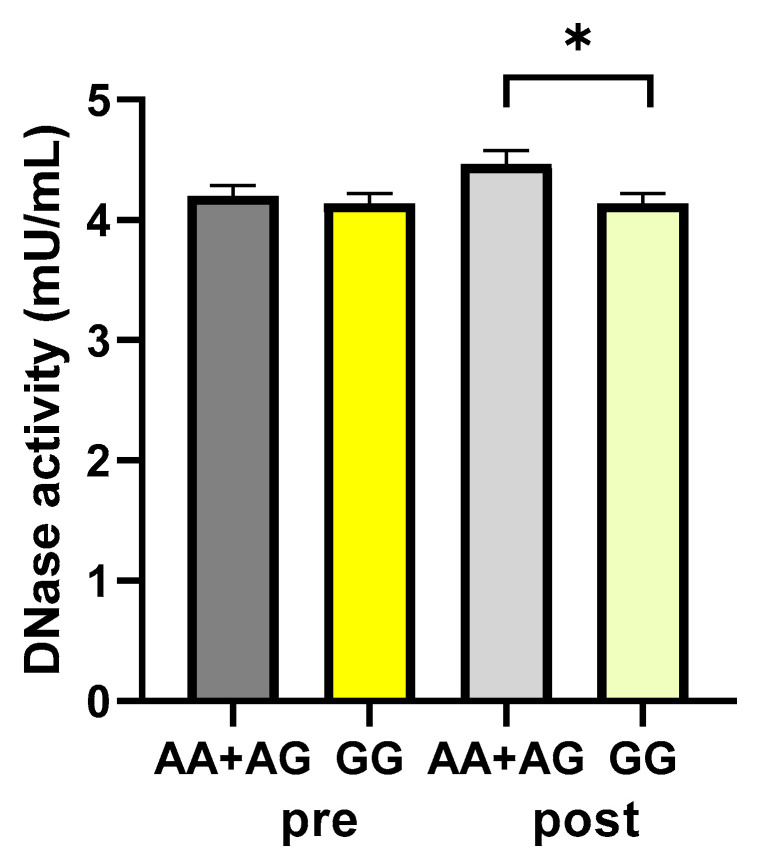
DNase activity according to the rs1053874 SNP. DNase activity was evaluated in 43 participants of the study, pre- and post-exercise program, dichotomized as carriers or not of the A allele (AA+AG and GG groups, respectively). * *p* < 0.05, unpaired *t*-test. Pre and post = before and after 12 weeks of the exercise program.

Subjects were categorized as carriers or not of the A allele (AA+AG and GG groups, respectively), which has been associated with higher-activity-harboring enzymes [31], and the DNase activity values were analyzed. According to the polymorphism, DNase activity values were not significantly different between the A carrier and non-A carrier groups, respectively, before the walking program (4.20 ± 0.08 vs. 4.14 ± 0.08 mU/mL, as A carrier and non-A carrier values, respectively; *p*: 0.5959, unpaired *t*-test). After exercise, a statistically significant difference was found between the carriers and non-carriers of the A allele, with values significantly higher in the A carriers (4.39 ± 0.11 vs. 4.08 ± 0.09 mU/mL, as A carrier and non-A carrier values, respectively; *p*: 0.0494, unpaired *t*-test) (Figure 6).

## 3. Discussion

Regular walking has been found to help patients with IBS by improving their psychological profile and reducing gastrointestinal symptoms [25,32,33].

Strong evidence supports the health benefits of PA, making it a common recommendation for promoting health and preventing health issues [24]. Regular PA reduces the risk of developing several chronic medical conditions and premature mortality, and international guidelines recommend at least 150 min per week of moderate to vigorous intensity PA [35].

Our previous study utilized a summary score of different field tests (GPCS) to explore the potential link between improved physical condition and reduced IBS symptoms. It demonstrated that improved IBS symptoms were effectively accompanied by increased GPCS (due to higher cardiorespiratory capacity, muscle mass, strength, endurance, and flexibility through the exercise program) [33].

As for the present study, data in the literature have shown that exercise can cause an increase in cf-DNA [29], due to factors such as oxygen deficiency, hyperlactatemia, and cytokine secretion, which increase the production of Reactive Oxygen Species (ROS) [26,27]. Hence, cf-DNA has been considered a molecular marker of inflammation and overtraining in exercise physiology [36]. cf-DNA changes have already been described in association with chronic exercise [37]. Some studies found a decline in cf-mtDNA in response to prolonged exercise [31], associated with increased DNase activity [30]. The health benefits of exercise may depend on the ability of muscles to interact with the inflammatory cells and stimulate or resolve inflammation to maintain immune homeostasis [38].

Among GI disorders, IBS represents a syndrome characterized by a low-grade inflammation [3,4]. Due to their central role in energy production, mitochondria are fundamental for maintaining intestinal barrier functions, so mitopathology is emerging in gut mucosal disorders. Molecules derived from damaged organella, mtDAMPs, constitute inflammatory signals underlying inflammatory cascade activation [11]. Considering this framework within the present study, we examined the response to prolonged moderate aerobic exercise of the highly immunogenic cf-mtDNA [15] and its endogenous counter-regulator, DNase [20], in IBS patients and compared it with the response in non-IBS healthy controls.

Altered regulation of an immune response resulting in the imbalanced levels of pro- and anti-inflammatory cytokines has been reported as having a role in the complex pathogenesis of IBS; increased circulating levels of the main inflammatory mediators in IBS, IL-6, IL-8 and TNF-alpha, and decreased level of IL-10 have been described [3]. Controlled intestinal inflammation is a natural feature that protects the gut from the massive load of microbes that reside in it and from food antigens. As such, in healthy subjects, IL-10 balances the inflammatory reactions to maintain immune homeostasis. We evaluated levels of these cytokines in the participants of this study. The profile in Table 2 shows a non-significant trend for increased circulating levels of the above-reported pro-inflammatory cytokines in IBS patients compared to non-IBS subjects at the baseline.

This scenario fits the low-grade inflammatory status and intrinsic heterogeneity of IBS [3]. The significantly reduced levels of the anti-inflammatory IL-10 in the patient group appear to confirm an altered capability of IBS patients to deal with inflammatory stimuli [3]. Therefore, as stated by other authors [3], and in accordance with our present findings, the persistent low-grade intestinal inflammation in individuals with IBS might stem from an imbalance in cytokine activity, potentially due to elevated pro-inflammatory cytokines or decreased anti-inflammatory cytokines. In this framework, boosting IL-10 levels could offer a promising treatment avenue for certain IBS patients, especially those with IBS-D.

On the other hand, the absence of a significant variation in cytokine levels after the intervention, which has been considered evidence of crosstalk between the exercising muscle and other organs [39], could be due to the moderate intensity and short PA duration adopted in this study.

Regarding cf-mtDNA, the results of the present study confirmed the high natural interindividual variation in the concentration of this molecule [36], without differences in this feature between the IBS patients and non-IBS subjects (Figure 3). Furthermore, as shown in Figure 4A, in accordance with data already reported by others [30,31,37], the results showed a reduction in cf-mtDNA in the non-IBS group, although not significant, as the adaptive response to prolonged exercise. Interestingly, the lowering of cf-mtDNA appeared to be associated with greater benefits in PC, as shown by the significantly higher GPCS values reached by subjects who experienced the reduction in circulating cf-mtDNA compared to those who experienced the gain. In the IBS group, this kind of adaptive response was not observed; in contrast, increased levels of cf-mtDNA were registered after the exercise program, so cf-mtDNA post-exercise values were significantly different between the two groups (Figure 4B). It appears IBS per se, with its low-grade chronic inflammation [3,4,40], likely influences cf-mtDNA levels, possibly enhancing its release or impairing its removal. Indeed, the interplay between oxidative stress/inflammation and dysfunctional mitochondria has been highlighted in animal models of IBS [41,42]. In addition, a marked increase in the level of plasmatic NET, as a by-product of inflammation-induced neutrophil activation, has also been reported in obese subjects, i.e., individuals who experience chronic low-grade inflammatory status [43].

Since cf-DNA can be kept low through its removal by DNases, enzymes that are secreted into body fluids to hydrolyze circulating DNA [44,45], the ability of the regular moderate PA to mediate the release of the waste-management DNases was investigated. Like cf-mtDNA, the DNase activity response to the exercise program differed between the two study groups, as shown in Figure 5. In the healthy control group, the significant inverse relationship between the cf-mtDNA levels and DNase activity suggests that the increased activity of the hydrolyzing enzyme, which acts as a protective factor against inflammation, may be able to maintain the homeostatic balance [13]. So, regular PA improves PC and raises the counteracting activity of DNase against pro-inflammatory signaling.

On the other hand, no significant rise in the DNase activity or correlation with the cf-mtDNA levels appeared in IBS patients after the exercise program. The present study suggests an imbalance between the release of cf-mtDNA and the enzymes that control its clean-up, which may reduce the health benefits of regular activity, at least as concerns the lowering of this pro-inflammatory molecule. The involvement of reduced circulating DNase activity in the inflamed bowel has already been demonstrated in IBD patients [45].

Since the *DNASE1* gene is polymorphic, genetic heterogeneity was investigated as a possible cause for the different enzyme responses between the two groups. The non-synonymous rs1053874 SNP was genotyped, as it has been described to be associated with enzyme activity [34]. The frequency of this SNP has been reported to differ among ethnicities [34,46]. In our Southern Italian samples, we found that the frequency of this SNP is similar to that of other Caucasian populations [34,46]. This polymorphism was previously evaluated in patients undergoing coronary intervention, and it was found that this genetic variant is associated with enzyme activity. However, no difference was observed in the frequency distribution between cases and controls, as shown in Table 3. Therefore, the rs1053874 SNP might be an independent predictor for decreased DNase activity [46]. The findings of this study align with those of Hofbauer et al. [46] and allow us to exclude a possible role for this polymorphism in the reported response to a twelve-week walking program in IBS patients.

## 4. Materials and Methods

### 4.1. Participants and Study Design

The Functional Gastrointestinal Disorders Research Group collaborated with the Laboratory of Movement and Wellness of the National Institute of Gastroenterology IRCCS “Saverio de Bellis” Castellana Grotte, BA, Italy, to recruit participants for their study. The project began in May 2022 and is still ongoing. The trial was registered on www.clinicaltrial.gov (accessed on 16 November 2023) with registration number NCT05453084.

This study is ancillary to the above-cited clinical trial, registered at www.clinicaltrial.gov (accessed on 16 November 2023). The study involved adults who met the Rome III–IV criteria for IBS, were referred by local general practitioners for gastrointestinal symptoms, or attended the Outpatient Clinic for Celiac Disease and Functional Disorders [47]. The participants had to be between 18 and 65 years old, be available to join the walking group, and have a medical certificate in non-competitive sports fitness. The exclusion criteria included individuals with serious cardiac, hepatic, metabolic, neurological, or psychiatric diseases, organic gastrointestinal disorders (e.g., inflammatory bowel diseases or autoimmune disorders), previous adherence to a low FODMAPs, vegan, or gluten-free diet, use of antidepressants, significant orthopedic or neuromuscular limitations, and absolute contraindications to exercise. Moreover, the analysis would not include subjects who missed more than 20% of their training sessions.

All procedures adhered to the guidelines set forth by the World Medical Association Declaration of Helsinki, the International Conference on Harmonization on Good Clinical Practice Guidelines, and the Ethical Conduct for Research Involving Humans [48]. The study group also followed the Consolidated Standards of Reporting Trials (CONSORT) guidelines for reporting on randomized clinical trials [49]. The trial received approval from the local Ethics Committee (Prot. N. 167/CE De Bellis).

### 4.2. Data Collection

At enrolment, all participants gave informed consent and completed a structured questionnaire on sociodemographic factors, lifestyle, and medical history. PA levels were assessed using the validated International Physical Activity Questionnaire, Short Form (IPAQ-SF) [50]. In addition, patients completed the Gastrointestinal Symptom Rating Scale (GSRS) to assess GI symptoms. Selected patients’ symptom intensity and frequency resembling IBS were evaluated using the IBS-Severity Scoring System (IBS-SSS), as previously reported [32,33].

Trained personnel collected fasting blood samples for biochemical valuations, conducted anthropometric measurements (weight, height, waist circumference), and performed bio-impedance analysis. The participants’ physical characteristics were assessed using different anthropometric parameters (height, weight, mid-upper arm, waist circumference, and hip circumferences). The BMI was also calculated (kg/m^2^). The measurements were taken using a SECA 700 mechanical column scale and a SECA 220 altimeter (INTERMED S.r.l., Milan, Italy).

**Figure 7:**
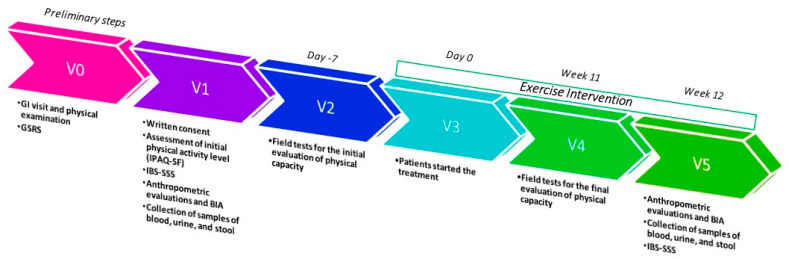
Study design timeline; GI = gastrointestinal; GSRS = Gastrointestinal Symptom Rating Scale; IBS-SSS = IBS-severity Scoring System; BIA = bioelectrical impedance analysis.

All measurements were taken at the beginning of the study and again after 12 weeks. The study’s timeline is illustrated in Figure 7.

### 4.3. Exercise Protocol

As previously described [32,33], three field tests assessed subjects’ baseline conditions and determined optimal training intensity. The tests included a 2 km walk for cardiorespiratory capacity, and Hand Grip and Sit-and-Reach tests for strength and flexibility. Participants received detailed instructions before the tests to ensure accuracy. Experts provided guidance and verified equipment functionality over three 60 min sessions preceding the tests.

Efforts were made to maintain consistency in test conditions as follows: (a) tests were conducted in the same location, (b) supervised by the same personnel, (c) at consistent times, and (d) using identical instruments wherever possible.

PA was organized into “Walking Groups” with the following structure: three weekly outdoor aerobic sessions on non-consecutive days for 12 weeks. The moderate intensity (60/75% of HR max) was monitored with heart rate monitors and personalized using Tanaka’s formula [51]. The rhythm and fatigue perceptions were assessed by the Talk Test [52] and Borg scale [53]. The walking ranged from 5 to 10 km/h, and each session lasted 60 min, totaling 180 min weekly. The session structure included a warm-up (5 min), normal walking (10 min), sustained walking (30 min), fast walking (10 min), and cool-down (5 min). Sessions were supervised by experts (Graduates in Preventive and Adapted Physical Activity Science and Techniques) with attendance meticulously recorded.

Physical capacity (PC) was assessed using motor tests of varying difficulty, that were validated for adults, to measure cardiorespiratory capacity, strength, and flexibility. A PC score was derived by combining test results, and each scored from 0 to 2 based on performance categories. Scores from the three tests were summed to generate an overall score ranging from 0 to 6 points. This approach, adapted from Bouchard et al. [54], offers a comprehensive measure of physical performance, integrating tasks which are relevant to daily activities.

### 4.4. Serum Cytokine Profiles and Isolation of cf-DNA from Plasma

All the analytical measurements were performed at the time of enrolment. The measurements were repeated at the end of the exercise program. A blood sample was collected after an overnight fast, immediately centrifuged and stored at −80 °C until further analysis.

Serum levels of interleukin-6 (IL-6), interleukin-8 (IL-8), TNF-alpha, and interleukin-10 (IL-10) were measured in duplicates using commercially available sandwich enzyme-linked immunosorbent assay kits (My BioSource, San Diego, CA, USA).

For human plasma processing, 12–18 mL of blood, with at least 9 mL, were collected in an ethylenediaminetetraacetic acid (EDTA) tube (Vacutainer, Becton Dickinson, Franklin Lakes, NJ, USA). The collected blood was processed within 2 h. The EDTA blood was centrifuged at 1000× *g* for 10 min at 4 °C, and the plasma fraction obtained was transferred to a 15 mL Falcon tube. Subsequently, it underwent further centrifugation at 3000× *g* for 10 min at 4 °C to eliminate platelets, yielding “cell-free plasma”. The plasma was divided into 0.5 mL aliquots and stored at −80 °C until further analysis.

Extracellular DNA fragments (cf-DNA) present in the bloodstream were isolated from 500 µL of cell-free plasma utilizing the column-based Plasma/Serum Circulating DNA Purification Mini Kit (Norgen Biotek Corp., Thorold, ON, Canada) as per the manufacturer’s protocol. Before processing, the frozen cell-free plasma samples underwent an additional centrifugation step at 400× *g* for 2 min. Due to the applied centrifugation force, the entire cell-free fraction of mt-DNA was expected to be preserved in the preparation [55]. The purified cf-DNA was eluted in 30 µL of nuclease-free water and subsequently utilized for ddPCR analysis.

### 4.5. cf-mtDNA Quantification

The concentration of cf-mtDNA (copies/µL) in plasma was quantified using a digital droplet PCR (ddPCR) assay, employing the Bio-Rad copy number determination assay on a QX200 ddPCR system. Specifically, the dHsaCNS669425578 assay targeted the NADH dehydrogenase 1 (*ND1*) mitochondrial DNA gene for copy number determination. In brief, a total reaction volume of 22 µL contained 4 µL of 10-fold diluted template DNA, 11 µL of 2× ddPCR Supermix for probes (without dUTPs), 0.55 µL of 20× ddPCR assays, and 6.45 µL of nuclease-free water. This mixture generated a minimum of 10,000 droplets using the Bio-Rad Automated Droplet Generator (Bio-Rad Laboratories Inc., Hercules, CA, USA). The PCR conditions were as follows: initial denaturation at 95 °C for 10 min, followed by 40 cycles of denaturation at 95 °C for 15 s and annealing/extension at 60 °C for 1 min (ramp rate: 2.5 °C per second), and a final extension at 98 °C for 10 min. Droplets were subsequently analyzed using the QX200 reader, and data were interpreted using the QX Manager Software 2.0 (Bio-Rad Laboratories Inc., Hercules, CA, USA).

### 4.6. Measurement of Serum DNase Activity

Total DNase activity in the serum samples was assessed using a single radial enzyme diffusion assay [30]. A brief overview of the procedure is as follows: (1) Calf thymus DNA (Sigma-Aldrich, St. Louis, MO, USA) was dissolved at a concentration of 100 µg/mL in assay buffer, which included 2.5 X GelRed (Sigma-Aldrich, St. Louis, MO, USA) as a DNA-binding fluorescent dye. (2) After heating the solution for 10 min at 50 °C, it was mixed with an equal volume of 2% agarose in water and solidified in a plastic tray. (3) Serum samples (2 µL) or dilutions of recombinant DNase I (Promega Corporation, Madison, WI, USA), known for their concentrations and used for creating the calibration curve, were loaded into the gel. (4) The gel was then incubated overnight at 37 °C. (5) The circles’ diameters representing the hydrolyzed DNA’s remaining fluorescence were measured using the Chemi Doc System and Image Lab 6.1software (Bio-Rad Laboratories Inc., Hercules, CA, USA).

### 4.7. Total DNA Extraction and Genotyping of the DNASE1 rs1053874 SNP by the PCR-RFLP Method

Peripheral blood mononuclear cells (PBMCs) were isolated from blood samples using density gradient sedimentation with Ficoll–Paque (GE Healthcare, Little Chalfont, UK). Total cellular DNA was extracted from 5 µL of isolated PBMCs using the QIAamp DNA Blood Mini Kit (QIAGEN, Hilden, Germany).

A genotyping assay for the rs1053874 non-synonymous SNP in the *DNASE1* gene was conducted using the PCR-RFLP (restriction fragment length polymorphism) method, as described by Yasuda et al. [34]. In brief, 20 ng of total DNA was amplified in a 20 µL reaction using the following primers: Forward 5′ CATCTGGGGATAAGAGGAGAG 3′ and Reverse 5′ AGTCGGGAACAACGGCGACT 3′, with an annealing temperature of 63 °C. The resulting amplicons were then digested with HinfI restriction endonuclease, and the digestion products (A allele: 247 bp; G allele: 227 bp) were separated using a 1.8% agarose gel.

### 4.8. Statistics

Subjects’ characteristics are described by mean ± SD. All data represent the results of at least two independent experiments and are expressed as mean ± SEM. Data were analyzed by unpaired *t*-test or Mann–Whitney test for comparing normal and non-normal distributed variables in unpaired samples, respectively, and paired *t*-test or Wilcoxon matched-pair signed-rank test for comparison in paired samples. Spearman’s correlation test was used for the correlation analysis. The allele and genotype frequencies at the rs1053874 SNP were estimated using gene counting. Differences in categorical variables, the polymorphic distribution between the study groups, and deviations from Hardy–Weinberg equilibrium were assessed using the χ2 test or Fisher’s exact test when appropriate. Statistical significance was established at *p* < 0.05. The statistical analysis was performed with Stata Statistical Software 18 (Corp, 4905 Lakeway Drive, College Station, TX, USA).

## 5. Conclusions

In conclusion, data from this study confirm the beneficial effects of regular exercise in increasing PC, and balancing cf-mtDNA release and clean-up by DNase activity, at least in non-IBS healthy subjects. This study improves the knowledge about the positive influence of PA, since it adds information regarding the impact of real-life moderate regular activity over a medium time period. As concerns the effectiveness of the exercise program under investigation as an alternative treatment of IBS, data shown here suggest that this intervention alone may not be sufficient in cases with persistent sub-clinical inflammation; instead, exercise may need to be paired with other treatments, such as biological and pharmaceutical compounds. However, a critical inquiry emerges as to whether the chosen observation period could be too short to allow for an improvement in the outcome under investigation in the IBS group, i.e., the concentration of one of the circulating pro-inflammatory molecules. Exploring this aspect through more prolonged protocols could be a fruitful avenue for future research.

## Figures and Tables

**Table 1:** Characteristics of participants at the baseline and after exercise intervention.

Exercise Intervention						
	Pre			Post		
	IBS (n. 26)	Non-IBS (n. 17)	*p*	IBS (n. 26)	Non-IBS (n. 17)	*p*
Sex (Male/Female)	6M/20F	3M/14F	0.9999 *			
Age (years)	55.8 ± 6.3	56.9 ± 5.8	0.5763			
BMI (kg/m^2^)	27.72 ± 3.8	28.29 ± 4.8	0.6731	27.26 ± 3.7	27.81 ± 4.8	0.6740
**IPAQ Categories**						
Inactive	10 (38.5%)	5 (29.4%)				
Sufficiently Active	10 (38.5%)	9 (53%)	0.6460 ^§^			
Active/Very Active	6 (23%)	3 (17.6%)				

IBS = irritable bowel syndrome; pre and post = before and after 12 weeks of the exercise program. BMI = Body Mass Index; IPAQ = International Physical Activity Questionnaire. IPAQ was categorized according to the metabolic equivalent of the task (MET) into inactive (<700 MET), sufficiently active (700–2519 MET), and active/very active (>2520 MET). Categorical data are represented as numbers; continuous data are reported as mean ± SD. *p*: unpaired *t*-test. *: Fisher’s exact test. ^§^: χ2 test. Statistical significance: *p* < 0.05.

**Table 2:** Cytokine profile of the participants at the baseline and after exercise intervention.

Exercise Intervention						
	Pre			Post		
	IBS (n. 26)	Non-IBS (n. 17)	*p*	IBS (n. 26)	Non-IBS (n. 17)	*p*
IL-6 (pg/dL)	1.48 ± 0.20	1.17 ± 0.11	0.8105	1.53 ± 0.24	1.25 ± 0.17	0.9657
IL-8 (pg/dL)	6.18 ± 0.70	5.52 ± 0.69	0.6272	7.78 ± 1.27	5.77 ± 1.22	0.4272
TNF-alpha (pg/dL)	4.81 ± 3.21	3.27 ± 1.27	0.7634	5.62 ± 3.41	3.85 ± 1.51	0.8973
IL-10 (pg/dL)	4.82 ± 0.97	7.58 ± 1.41	0.04776	4.61 ± 0.83	6.68 ± 1.66	0.0818

Data are expressed as mean ± SEM. *p* = Mann–Whitney test. Statistical significance *p* < 0.05. IBS = irritable bowel syndrome; pre and post = before and after 12 weeks of the exercise program.

**Table 3:** Genotype distribution and allele frequencies at the rs1053874 SNP in the two study groups.

	IBS (n = 26)	Non-IBS (n = 17)	*p*
GG	10 (0.39)	7 (0.41)	0.9429
AG	12 (0.46)	8 (0.47)	
AA	4 (0.15)	2 (0.12)	
G	0.62	0.65	0.8223 *
A	0.38	0.35	

The absolute number of genotypes and frequencies (in parentheses) are reported. *p*: χ2 test, * Fisher’s exact test. IBS = irritable bowel syndrome.

## Data Availability

Data are available upon reasonable request.
